# Development of Modified Japanese Versions of Questionnaires to Assess Physical and Cognitively Stimulating Activities

**DOI:** 10.31662/jmaj.2024-0153

**Published:** 2025-03-07

**Authors:** Shoya Matsumoto, Tomomi Satoh, Mitsuru Shinohara, Reo Kawano, Keisuke Suzuki, Janina Krell-Roesch, Michio Ide, Hirotaka Watada, Masahiro Akishita, Hidenori Arai, Izumi Kondo, Yonas E. Geda, Naoyuki Sato

**Affiliations:** 1 Department of Aging Neurobiology, Center for Development of Advanced Medicine for Dementia, National Center for Geriatrics and Gerontology, Obu, Japan; 2 Department of Geriatric Medicine, Graduate School of Medicine, The University of Tokyo, Tokyo, Japan; 3 Department of Aging Neurobiology, Graduate School of Medicine, Osaka University, Suita, Japan; 4 Innovation Center for Translational Research, National Center for Geriatrics and Gerontology, Obu, Japan; 5 Institute of Sports and Sports Science, Karlsruhe Institute of Technology, Karlsruhe, Germany; 6 Nishiki Memorial Hospital, Tamba-Sasayama, Japan; 7 Department of Metabolism and Endocrinology, Graduate School of Medicine, Juntendo University, Tokyo, Japan; 8 Tokyo Metropolitan Institute for Geriatrics and Gerontology, Tokyo, Japan; 9 National Center for Geriatrics and Gerontology, Obu, Japan; 10 Department of Neurology and the Franke Global Neuroscience Education Center, Barrow Neurological Institute, Phoenix, USA

**Keywords:** questionnaire, physical activities, cognitively stimulating activities, lifestyle, reliability

## Abstract

**Introduction:**

Lifestyle factors such as physical and cognitively stimulating activities may protect against various diseases. However, only a few simple and validated questionnaires assess the lifestyle factors in Japan. Thus, we aimed to create Japanese versions of such questionnaires for assessing physical and cognitively stimulating activities. This study examined their inter-rater reliability and test-retest reproducibility.

**Methods:**

We developed a Japanese version of questionnaires by translating the English questionnaire that assesses the frequency of several physical and cognitively stimulating activities. Additionally, the Japanese version assesses the duration of engagement in physical activities, and we have added mental activities such as meditation and Zen practice. The inter-rater reliability and test-retest reproducibility of evaluating the frequency, duration, frequency × duration of each physical activity, and frequency of each cognitively stimulating activity were tested in healthy volunteers.

**Results:**

The study included 48 participants aged 25-67 years. We observed good inter-rater reliability and test-retest reproducibility for the physical and cognitively stimulating activity questionnaires. As a pilot approach, we calculated the Total Physical Activity Score (metabolic equivalents·min/week) with an intraclass correlation coefficient (ICC) (2,1) of 0.818 (95% confidence interval, 0.698-0.894), indicating good test-retest reproducibility.

**Conclusions:**

The Japanese versions of questionnaires used to assess the frequency and duration of physical and cognitively stimulating activities generally have good inter-rater reliability and test-retest reproducibility. While introducing the duration of engagement might enable the estimation of the Total Physical Activity Score, further validation using objective measures of activities and other self-reported physical activity questionnaires is necessary, which is a limitation of this study.

## Introduction

Lifestyle factors, such as physical and cognitively stimulating activities may impact various diseases, including geriatric diseases such as dementia, frailty, and vascular diseases ^(1), (2), (3), (4), (5), (6)^. Thus, surveys with validated questionnaires that measure lifestyle factors are critical proximal steps to potentially prevent diseases and promote health. However, only a few simple and validated questionnaires assess lifestyle factors in Japan^(7), (8), (9), (10)^. Several questionnaires assessing the frequency of physical and cognitively stimulating activities were developed in English ^(11), (12)^ and have been used in research for examining the associations between lifestyle factors and the risk of mild cognitive impairment or dementia ^(13), (14), (15), (16), (17), (18), (19)^. Creating a Japanese version of the English questionnaires would enable future comparisons among studies conducted in different countries. The International Physical Activity Questionnaire (IPAQ) and the Global Physical Activity Questionnaire, GPAQ are available in Japanese and are used in research ^(20), (21)^; these questionnaires assess only the physical activities lasting for >10 min ^(9), (10)^. Although the Japan Arteriosclerosis Longitudinal Study Physical Activity Questionnaire was initially developed and well-validated in Japan, assessing physical activities lasting <10 min ^(22)^, its use in international collaborative studies may be challenging because there is no English version. The Japan Public Health Center-based Prospective Study Questionnaire compactly assesses the physical activities but not time ^(23)^.

Lifestyle factors substantially vary among different countries and cultures. Thus, creating culturally congruent questionnaires that match a country’s or cultural area’s lifestyle habits is necessary. Therefore, this study aimed to create Japanese versions of questionnaires used in the US to assess the frequency of engagement in physical and cognitively stimulating activities effectively and evaluate the Japanese version of the physical and cognitively stimulating activities questionnaires by examining the inter-rater reliability and test-retest reproducibility.

## Materials and Methods

### Development of a modified Japanese version of the physical and cognitively stimulating activity questionnaire

We translated two validated questionnaires used in the Mayo Clinic Study of Aging (MCSA) in Olmsted County, Minnesota, USA, for assessing the frequency of physical and cognitively stimulating activities in Japanese. The questionnaires were derived from two validated instruments, the 1985 National Health Interview Survey and the Minnesota Heart Survey intensity codes ^(24), (25)^. Three levels of physical activity and exercise intensity are distinguished by providing examples for each level: light physical activity (e.g., laundry, vacuuming, making beds, or dusting), moderate physical activity (e.g., scrubbing floors, washing windows, gardening, or raking leaves), heavy physical activity (e.g., carrying heavy objects, heavy digging, pushing a mower, or hard manual labor), light physical exercise (e.g., leisurely walking or slow dancing), moderate physical exercise (e.g., hiking or swimming), and vigorous physical exercise (e.g., jogging or playing singles tennis). In addition, participants were asked to indicate the frequency at which they performed these activities: ≤1 times per month, 2-3 times per month, 1-2 times per week, 3-4 times per week, 5-6 times per week, and daily. In the MCSA, the questionnaires are used to assess the physical activities performed by the participants during two time periods, i.e., in the last 12 months and when the participants were 50-65 years old. Reportedly, the physical activity questionnaire has moderate to good internal consistency, and the test-retest correlation coefficients was 0.33 for vigorous intensity activity and 0.50 for moderate intensity activity ^(11)^. Similar to the physical activity questionnaire, the questionnaire assessing cognitively stimulating activities inquired engaging in reading books, craft activities (e.g., knitting, quilting), computer use, playing games (e.g., puzzles, crosswords), and social activities (e.g., going out to movies and theaters) and assessed the frequency of engagement using the same categories as in the physical activities questionnaire (i.e., ≤1 times per month, 2-3 times per month, 1-2 times per week, 3-4 times per week, 5-6 times per week, and daily) ^(18)^. Several papers have been published based on both questionnaires^(11), (12), (16), (18)^.

Because the lifestyle is different in Japan and the US, simply translating the English questionnaire into Japanese would hinder its widespread use. Therefore, we have made modifications to accommodate the differences in lifestyles between Japan and the US. The US version does not include items on the duration of engagement in physical and cognitively stimulating activities, making it difficult to quantitatively evaluate physical activity and exercise, e.g., by calculating energy expenditure using metabolic equivalents (METs). Therefore, we added an item to estimate the duration of physical activities. Furthermore, the frequencies of performing physical and cognitively stimulating activities in the US questionnaire were assessed through six categories, i.e., ≤1 times per month, 2-3 times per month, 1-2 times per week, 3-4 times per week, 5-6 times per week, and every day ^(11)^. As the Japanese way of thinking does not include “zero or none,” we created two separate categories of “none” and “≤1 per month” in the Japanese version. Finally, a new item on mental activities, such as meditation and Zen practice, was introduced in the questionnaire to assess the frequency of cognitively stimulating activities.

Two geriatricians translated the English questionnaires into Japanese, and two additional senior geriatricians reviewed the Japanese version. Next, the Japanese version was modified as described above. The back translation was performed by a Japanese translator―who graduated from a top-ranked University with >10 years of experience and EIKEN 1^st^ grade (highest), which is Japan’s most widely recognized English language assessment―and double-checked by a native speaker who lived in North America. The back translation of examples of physical and cognitively stimulating activities is described in the Supplementary Methods and shown in Supplementary Figure 1 and 2 respectively.

### Study protocol and participants

The Clinical Research Ethics Committee of the National Center for Geriatrics and Gerontology approved this study (No. 1590-4). This study conforms to the provisions of the Declaration of Helsinki (revised in Tokyo in 2004). We examined the inter-rater reliability and test-retest reproducibility of the Japanese versions of the physical and cognitively stimulating activity questionnaires. A total of 30 Obu City employees, 10 Nishiki Memorial Hospital workers, and 8 Nakano Gastrointestinal Hospital workers provided written informed consent and participated in this study. The anonymity of the participants was also preserved. The participants were healthy volunteers. First, under the guidance of examiner A, the participants completed two questionnaires to assess physical and cognitively stimulating activities (Supplementary Figure 3 and 4). The examiner explained the general contents of the questionnaires to the participants and, while resolving doubts, if any, asked them to fill the questionnaires. The participants completed the same questionnaires on the same day under the guidance of examiner B. After 2 weeks, the participants completed the questionnaires under the guidance of examiner A. The inter-rater reliability was assessed by comparing the answers provided under examiner A’s and B’s guidance on day 1. The test-retest reproducibility was assessed by comparing the answers on day 1 and 2 weeks later, under the guidance of examiner A.

### Total physical activity score

**Figure 1.**
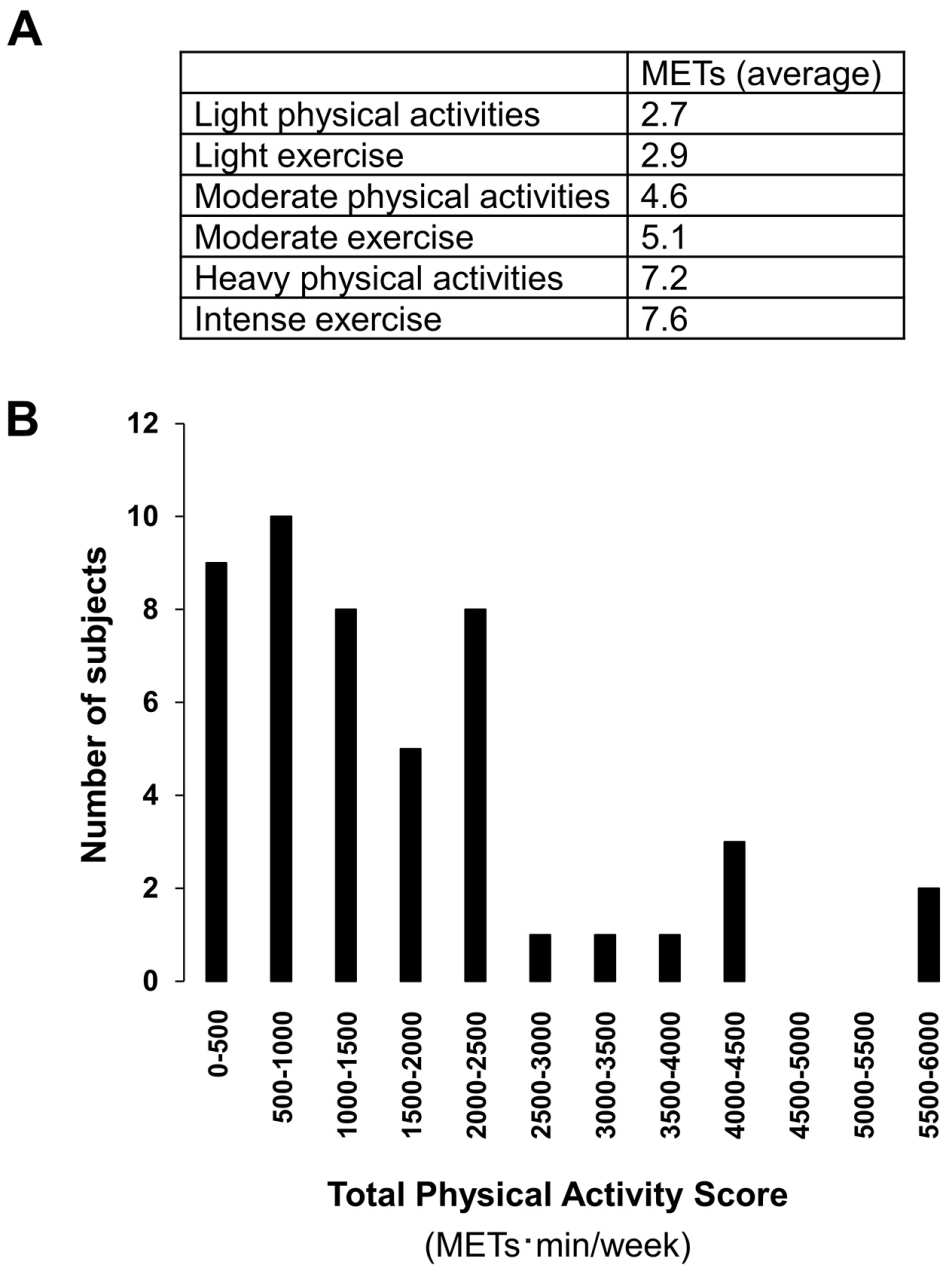
Total Physical Activity Scores A. Averaged METs of physical activities for calculating Total Physical Activity Score B. A histogram of Total Physical Activity Scores (METs·min/week) of 48 participants calculated as 2.7 (METs) × frequency of light physical activity (/week) × duration (min) + 2.9 (METs) × frequency of light exercise (/week) × duration (min) + 4.6 (METs) × frequency of moderate physical activity (/week) × duration (min) + 5.1 (METs) × frequency of moderate exercise (/week) × duration (min) + 7.2 (METs) × frequency of heavy physical activity (/week) × duration (min) + 7.6 (METs) × frequency of intense exercise (/week) × duration (min).

We calculated a Total Physical Activity Score (METs·min/week) using the physical activity questionnaire (Figure 1A). The Total Physical Activity Score reflects the sum of the METs for each self-reported physical activity or exercise multiplied by frequency per week (i.e., 0, ≤1 times per month, 2-3 times per month, 1-2 times per week, 3-4 times per week, 5-6 times per week, and every day) and duration per day when physical activity or exercise was performed (min). METs indicate physical activity intensity by estimating how much energy is consumed compared with resting (which is set at 1 MET) ^(26)^. MET values for each activity in the physical activity questionnaire are shown in Supplementary Table 1. Reportedly ^(11)^, the scores to indicate the frequency of engaging derived from the six response categories are 0 for none, 0.1 for ≤1 times per month, 0.5 for 2-3 times per month, 1.5 for 1-2 times per week, 3.5 for 3-4 times per week, 5.5 for 5-6 times per week, and 7 for daily. Thus, the formula for the Total Physical Activity Score (METs·min/week) was 2.7 (METs) × frequency of light physical activities (/week) × duration (min) + 2.9 (METs) × frequency of light exercise (/week) × duration (min) + 4.6 (METs) × frequency of moderate physical activities (/week) × duration (min) + 5.1 (METs) × frequency of moderate exercise (/week) × duration (min) + 7.2 (METs) × frequency of heavy physical activities (/week) × duration (min) + 7.6 (METs) × frequency of intense exercise (/week) × duration (min). Next, we examined the inter-rater reliability and test-retest reproducibility of the Total Physical Activity Score of each participant.

### Statistics

We performed statistical analyses using JMP Pro software (version 13, SAS Institute Inc.) or Excel 2021 (Microsoft Corp.). We calculated Cronbach’s alpha and Spearman’s rank correlation coefficients to examine the inter-rater consistency between two examiners, and intraclass correlation and Spearman’s rank correlation coefficients to examine the reproducibility of test-retest between the two testing sessions (in an interval of 2 weeks) ^(27)^. Intraclass correlation coefficient (ICC) estimates and their 95% confidence intervals (CIs) were calculated based on a two-way random-effects model, single measurement, and absolute agreement (ICC(2.1)).

Based on the 95% CI of the ICC estimate, values <0.5, 0.5-0.75, 0.75-0.9, and >0.9 indicate poor, moderate, good, and excellent reliability, respectively ^(27)^. We considered *p* < 0.05 as statistically significant.

## Results

The study included 48 participants aged 25-67 years (mean ± SD age, 49.8 ± 11.7 years; 28 men and 20 women). The results of the inter-rater reliability of the physical activity questionnaire are summarized in Table 1 (left side). The Cronbach’s alpha coefficients for the frequencies/durations/and frequency × duration were as follows: light physical activity (0.971/0.955/0.951), light exercise (0.977/0.982/0.982), moderate physical activities (0.969/0.963/0.996), moderate exercise (0.972/0.914/0.985), heavy physical activity (0.838/0.948/0.997), and intense exercise (0.979/0.975/0.995). The Spearman’s rank correlations also agreed with the results of the Cronbach’s alpha coefficient analyses. Overall, these results showed good inter-rater reliability concerning frequency, duration, and frequency × duration of the physical activity questionnaire.

**Table 1.** Inter-Rater Reliability and Test-Retest Reproducibility of the Physical Activity Questionnaire.

		Inter-rater reliability			Test-retest reproducibility		
		Cronbach’s alpha coefficient	Spearman’s rank correlation		ICC (2,1)	Spearman’s rank correlation	
			rho	*p*-value		rho	*p*-value
Frequency	Light physical activity	0.971	0.944	<0.0001	0.847 (0.743, 0.912)	0.825	<0.0001
	Light exercise	0.977	0.972	<0.0001	0.854 (0.753, 0.916)	0.853	<0.0001
	Moderate physical activity	0.969	0.744	<0.0001	0.933 (0.883, 0.962)	0.704	<0.0001
	Moderate exercise	0.972	0.964	<0.0001	0.684 (0.498, 0.810)	0.819	<0.0001
	Heavy physical activity	0.838	0.953	<0.0001	0.792 (0.656, 0.878)	0.822	<0.0001
	Intense exercise	0.979	0.905	<0.0001	0.379 (0.113, 0.595)	0.576	<0.0001
Average duration per day (min)	Light physical activities	0.955	0.904	<0.0001	0.874 (0.786, 0.928)	0.827	<0.0001
	Light exercise	0.982	0.906	<0.0001	0.930 (0.879, 0.960)	0.823	<0.0001
	Moderate physical activity	0.963	0.836	<0.0001	0.850 (0.747, 0.913)	0.600	<0.0001
	Moderate exercise	0.914	0.896	<0.0001	0.762 (0.611, 0.859)	0.774	<0.0001
	Heavy physical activity	0.948	0.951	<0.0001	0.205 (−0.084, 0.462)	0.786	<0.0001
	Intense exercise	0.975	0.889	<0.0001	0.768 (0.623, 0.863)	0.596	<0.0001
Frequency × Duration	Light physical activity	0.951	0.894	<0.0001	0.861 (0.765, 0.920)	0.818	<0.0001
	Light exercise	0.982	0.955	<0.0001	0.576 (0.348, 0.739)	0.824	<0.0001
	Moderate physical activity	0.996	0.793	<0.0001	0.990 (0.983, 0.995)	0.749	<0.0001
	Moderate exercise	0.985	0.968	<0.0001	0.965 (0.938, 0.980)	0.824	<0.0001
	Heavy physical activity	0.997	0.953	<0.0001	0.385 (0.112, 0.602)	0.802	<0.0001
	Intense exercise	0.995	0.913	<0.0001	0.576 (0.353, 0.737)	0.583	<0.0001
Regular programs		1	1	1	<0.0001	1 (1, 1)	1

ICC (2, 1) is presented as the coefficient with a 95% confidence interval. ICC, intraclass correlation coefficient

The results of the test-retest reproducibility of the physical activity questionnaire are summarized in Table 1 (right side). The ICC (2,1) values for the frequencies/durations/and frequency × duration were as follows: light physical activity (0.847/0.874/0.861), light exercise (0.854/0.930/0.576), moderate physical activities (0.933/0.850/0.990), moderate exercise (0.684/0.762/0.965), heavy physical activity (0.792/0.205/0.385), and intense exercise (0.379/0.768/0.576). Moreover, all items were statistically significant in the Spearman’s rank correlation analyses. Overall, the test-retest reproducibility for the frequency, duration, and frequency × duration of the physical activity questionnaire had moderate to excellent reliabilities, except for duration and frequency × duration of heavy physical activities and the frequency of intense exercise, although the Spearman’s rank correlations were statistically significant.

**Figure 2.**
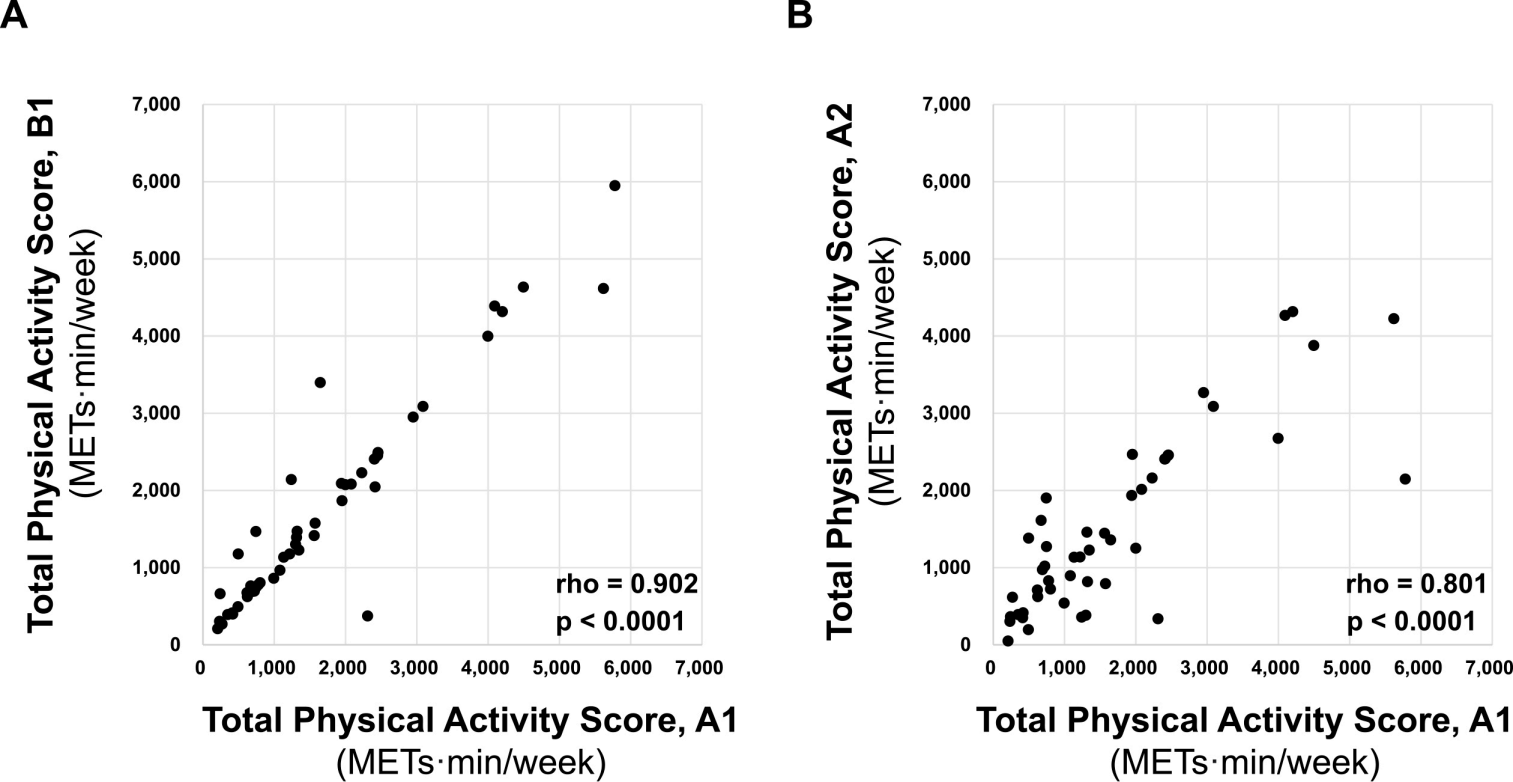
Spearman’s rank correlation between inter-rater reliability and test-retest reproducibility of Total Physical Activity Score Inter-rater reliability (A) and test-retest reproducibility (B) of Total Physical Activity Score were well retained (rho = 0.902, *p* < 0.0001, and rho = 0.801, *p* < 0.0001, respectively). A1: examiner A, 1^st^; B1: examiner B, 1^st^; A2: examiner A, 2^nd^.

Figure 1B displays the distribution of Total Physical Activity Score (METs·min/week) using the physical activity questionnaire. The Cronbach’s alpha coefficient for the inter-rater reliability of the Total Physical Activity Score was 0.971; the ICC (2,1) value to indicate test-retest reproducibility was 0.818 (95% CI, 0.698-0.894). Moreover, the Spearman’s rank correlation showed that the inter-rater reliability and test-retest reproducibility of the score were well retained (r = 0.902, *p* < 0.0001, and r = 0.801, *p* < 0.0001, respectively) (Figure 2A and B). While analyzing >55 years old participants (n = 18), we observed good inter-rater reliability and test-retest reproducibility (Supplementary Figure 6). Additionally, when separated by sex, there were still good inter-rater reliability and test-retest reproducibility (Supplementary Figure 7). Overall, these results indicate good inter-rater reliability and test-retest reproducibility for the Total Physical Activity Score.

Finally, the Cronbach’s alpha coefficients to indicate the inter-rater reliability for the frequencies of reading newspapers, reading magazines, reading books, playing games, playing music by yourself, artistic activity, mental activity, handicraft, group activity, social activity, using a computer (including smartphone), and television were 0.990, 0.929, 0.928, 0.930, 0.926, 0.999, 1.000, 0.929, 0.884, 0.873, 0.898, and 0.986, respectively (Table 2). The Spearman’s rank correlation coefficients indicating the inter-rater reliability of the cognitively stimulating activity questionnaire were good. The ICC (2,1) to indicate test-retest reproducibility for the frequencies of reading newspapers, reading magazines, reading books, playing games, playing music by yourself, artistic activity, mental activity, handicraft, group activity, social activity, using a computer (including smartphone), and television were 0.974, 0.904, 0.857, 0.823, 0.947, 1, 0.994, 0.994, 0.737, 0.668, 0.667, and 0.862, respectively (Table 2). These results indicate that the test-retest reproducibility for the cognitively stimulating activities was moderate to excellent. Overall, these data suggest that the questionnaire used to assess cognitively stimulating activities had good inter-rater reliability and test-retest reproducibility.

**Table 2.** Inter-Rater reliability and Test-Retest Reproducibility of the Cognitively Stimulating Activity Questionnaire.

	Inter-rater reliability			Test-retest reproducibility		
	Cronbach's alpha coefficient	Spearman's rank correlation		ICC (2,1)	Spearman's rank correlation	
		rho	*p*-value		rho	*p*-value
Reading newspapers	0.990	0.978	<0.0001	0.974 (0.954, 0.985)	0.979	<0.0001
Reading magazines	0.929	0.844	<0.0001	0.904 (0.835, 0.945)	0.816	<0.0001
Reading books	0.928	0.922	<0.0001	0.857 (0.759, 0.917)	0.857	<0.0001
Playing games	0.930	0.985	<0.0001	0.823 (0.706, 0.897)	0.871	<0.0001
Playing music by yourself	0.926	0.846	<0.0001	0.947 (0.907, 0.970)	0.846	<0.0001
Artistic activities	0.999	1.000	<0.0001	1 (1, 1)	1.000	<0.0001
Mental activities	1.000	0.937	<0.0001	0.994 (0.989, 0.996)	1.000	<0.0001
Handicraft	0.929	0.877	<0.0001	0.994 (0.990, 0.997)	0.899	<0.0001
Group activities	0.884	0.937	<0.0001	0.737 (0.577, 0.843)	0.691	<0.0001
Social activities	0.873	0.723	<0.0001	0.668 (0.475, 0.800)	0.743	<0.0001
Using a computer (including smartphone)	0.898	0.939	<0.0001	0.667 (0.478, 0.798)	0.773	<0.0001
Television	0.986	0.927	<0.0001	0.862 (0.767, 0.920)	0.889	<0.0001

ICC (2, 1) is presented as the coefficient with a 95% confidence interval. ICC, intraclass correlation coefficient

## Discussion

We obtained good inter-rater reliability and test-retest reproducibility for the physical and cognitively stimulating activity questionnaires. Furthermore, as adding the duration of each session to the physical activity questionnaire helped estimate the total amount of physical activity, we calculated a Total Physical Activity Score using METs and observed its good inter-rater reliability and test-retest reproducibility.

Possible uses of these questionnaires include research (e.g., epidemiological, observational, intervention, and clinical studies), general practice, health checkups, individual use, and national and local government use, while these questionnaires still need some validation in Japanese. The questionnaires can be retrieved from the corresponding author upon request. The Japanese versions are also provided as supplemental materials. The questionnaires can also be used in general hospitals for everyday medical care and serve as a basis for communication between patients and clinicians. Obtaining information on patients’ engagement in physical and cognitively stimulating activities using these questionnaires can help clinicians provide feedback to the patients (e.g., on healthy lifestyle choices). The questionnaires can also be used for health checkups, for example, in middle-aged individuals, and for repeated measurements to track changes in patients’ engagement in physical and cognitively stimulating activities over time. Individual use may facilitate self-evaluation and reflection. Moreover, the questionnaires may be useful for citizen surveys by national and local governments or other stakeholders to inform policies or guidelines. Finally, investigating lifestyle factors over the course of a lifetime may prevent and treat various diseases ^(28), (29), (30), (31), (32), (33)^.

The limitations of this study are the small sample size and the convenience sample of healthy volunteer participants, in addition to the study population being biased. The participants’ ages were 25-67 years. Their occupational backgrounds were also skewed, with a high proportion of healthcare workers. Furthermore, the validity of the Total Physical Activity Score was not assessed. Future research is critically needed to examine the correlation between the Total Physical Activity Score obtained using this questionnaire and objective measures of physical activities, such as doubly labeled water ^(8)^ or accelerometry, to further validate the physical activity questionnaire.

A standard protocol for calculating total energy expenditure using several physical activity questionnaires used in Japanese cohort studies is also under consideration ^(34)^. GPAQ and IPAQ measure physical activities and exercises only when performed for >10 min ^(9), (10)^. The physical activity questionnaire provided herein inquires any physical activity and exercise, including those performed for <10 min, and thus might contribute to establishing this standard protocol. Another strength of this study is that we added “mental activities” such as “meditation,” “Zen―a Japanese form of Buddhism,” “prayer,” and “sutra chanting,” to the Japanese version of the questionnaire on the frequency of cognitively stimulating activities, even though these items were not included in the US questionnaire. Therefore, the Japanese version of the questionnaire is suitable to the Japanese culture, potentially contributing to the widespread use of this questionnaire in Japan.

In conclusion, the Japanese questionnaires used to assess physical and cognitively stimulating activities have good inter-rater reliability and test-retest reproducibility. The physical activity questionnaire requires further validation using objective measures of activities and other self-reported physical activity questionnaires. Because we have made culturally congruent modifications, such as adding mental activities including meditation and Zen practice, it will be helpful for Asians and other countries to create their national language versions based on this Japanese version.

## Article Information

### Conflicts of Interest

None

### Sources of Funding

This work was supported in part by the Research Funding for Longevity Sciences from NCGG (19-3 & 21-12 to NS).

### Acknowledgement

We thank Ms. Mika Kitagawa, Mr. Hiroshi Nakamura, and the study participants at the Obu City Municipal Office, Nishiki Memorial Hospital, and Nakano Gastrointestinal Hospital for their contribution. We express our gratitude to the members of the Department of Aging Neurobiology, the Innovation Center for Translational Research in the National Center for Geriatrics and Gerontology, the staff of Nishiki Memorial Hospital and Nakano Gastrointestinal Hospital for their help, and Dr. Yoshihiko Naito of Mukogawa Women’s University for the helpful discussions.

### Author Contributions

Conceptualization: NS and YEG; Data curation: NS, SM, TS, and MS; Formal analysis: NS, TS, MS, RK, and KS; Funding acquisition: NS; Investigation: NS, SM, TS, MS, and MI; Methodology: NS, SM, MS, JKR, HA, IK, and YEG; Supervision: HW, MA, HA, IK, and YEG; Writing: NS, SM, MS, JKR, and YEG. Shoya Matsumoto,Tomomi Satoh and Mitsuru Shinohara contributed equally to this work.

### Disclaimer

Masahiro Akishita is one of the Editors of JMA Journal and on the journal’s Editorial Staff. He was not involved in the editorial evaluation or decision to accept this article for publication at all.

## Supplement

Supplementary Materials
