# Supplementary material for: Development of Modified Japanese Versions of Questionnaires to Assess Physical and Cognitively Stimulating Activities
Source: JMA J. 2025 Mar 7;8(2):498–505. doi: 10.31662/jmaj.2024-0153 (PMC12095128; doi:10.31662/jmaj.2024-0153)
Supplement: Supplementary Materials [file 2433-3298-8-2-0498-s001.pdf]

Supplemental Materials

Table of Contents

Supplementary Methods. Back translation of examples of physical activities and exercise and of cognitively stimulating activities 2

Supplementary Figure 1. Back translation of modified Japanese version of physical activity questionnaires 4

Supplementary Figure 2. Back translation of modified Japanese version of cognitively stimulating activity questionnaires 5

Supplementary Figure 3. Distributions of frequencies of physical activity and exercise of thirty participants in this study (Examiner A, 1st) 6

Supplementary Figure 4. Distributions of frequencies of cognitively stimulating activity of thirty participants in this study (Examiner A, 1st) 7

Supplementary Figure 5. Individual Physical Activity Scores of participants 8

Supplementary Figure 6. Spearman rank correlation of Inter-rater reliability and test-retest reproducibility of "Total Physical Activity Score" of subjects over 55 years old 9

Supplementary Figure 7. Spearman rank correlation of Inter-rater reliability and test-retest reproducibility of "Total Physical Activity Score" of subjects stratified by female and male.. 10

Supplementary Table 1. MET values for each activity on the physical activity questionnaire 11

Supplementary material. The Japanese version of the questionnaires and FAQ 14

Supplementary Methods

Back translation of examples of physical activities and exercise and of cognitively stimulating activities

Back translation of examples of physical activities and exercise are as follows (Supplementary Figure 1): Light physical activities include washing, house cleaning, kitchen work, bed making, garbage disposal, home maintenance, and simple repair (including car). Light exercise includes slow walking, stretching, golfing with a cart, practice on the golf driving range, bowling, and slow ballroom dancing including waltzing. Moderate physical activities include gardening, DIY, window wiping, light snow shoveling, digging a sandbox, moving furniture, and wall painting. Moderate exercise includes fast walking, hiking, muscle-building, golfing without a cart, swimming, table tennis, playing doubles tennis, moderate use of exercise machines, yoga, and hula dance. Heavy physical activities include carrying heavy items, full-scale farming work, pushing a manual lawn mower, shoveling humid and heavy snow, and heavy physical labor. Intense exercise includes jogging, mountain climbing, playing singles tennis, soccer, skiing, riding a bicycle up a mountain path, and intense use of exercise machines.

Examples of cognitively stimulating activities are as follows (Supplementary Figure 2): Reading newspapers includes Yomiuri, Asahi, Mainichi, Sankei, Chunichi, Hokuriku, or Nihon Keizai Shimbun. Reading magazines includes business, sports, art (music, picture, etc.), or hobby (fishing, handicraft, etc.) magazines. Reading books includes novels and nonfiction books. Playing games includes card game, trump (playing cards), crossword puzzle, sudoku, igo, shogi, Othello, and other board games. Playing music by yourself includes a musical instrument, singing in a chorus or in a karaoke box, and playing music in an orchestra or a band. Artistic activities include painting with watercolors or oils, carving, and calligraphy. Mental activities include meditation, Zen practice, prayer, and sutra chanting. Handicraft includes woodworking, pottery, quilting, and sewing. Group activities include reading session, study meeting, and activities in local community. Social activities include going to a theater, movie, concert, watching a Japanese traditional storytelling including rakugo or bunraku, going out with friends,

and travelling. Using a computer (including smartphone) includes using the Internet, sending and receiving e-mails, playing computer games, searching on the web, and online shopping. The question regarding television is “how many hours per day do you watch TV?”

Supplementary Figure 1. Back translation of modified Japanese version of physical activity questionnaire

Physical activity

Number：
Date：

Please answer the following questions about your current physical activities.

For each of the physical activities and exercises shown below, please indicate your average frequency of participation for the past 12 months using the ☑ mark. In addition, please describe the average daily duration of your physical activities and exercise.							
**Light physical activities**<br>**Examples:** washing, house cleaning, kitchen work, bed making, garbage disposal, home maintenance or simple repair (including car)	None ☐	Once a month or less ☐	2-3 times /month ☐	1-2 times /week ☐	3-4 times /week ☐	5-6 times /week ☐	Every day ☐
		Average duration per day: ( ) minutes					
**Light exercise**<br>**Examples:** slow walking, stretching, golfing with a cart, practice on the golf driving range, bowling, slow ballroom dancing including waltzing	None ☐	Once a month or less ☐	2-3 times /month ☐	1-2 times /week ☐	3-4 times /week ☐	5-6 times /week ☐	Every day ☐
		Average duration per day: ( ) minutes					
**Moderate physical activities**<br>**Examples:** gardening, DIY, window wiping, light snow shoveling, digging a sandbox, moving furniture, wall painting	None ☐	Once a month or less ☐	2-3 times /month ☐	1-2 times /week ☐	3-4 times /week ☐	5-6 times /week ☐	Every day ☐
		Average duration per day: ( ) minutes					
**Moderate exercise**<br>**Examples:** fast walking, hiking, muscle-building, golfing without a cart, swimming, table tennis, playing doubles tennis, moderate use of exercise machines, yoga, hula dance	None ☐	Once a month or less ☐	2-3 times /month ☐	1-2 times /week ☐	3-4 times /week ☐	5-6 times /week ☐	Every day ☐
		Average duration per day: ( ) minutes					
**Heavy physical activities**<br>**Examples:** carrying heavy items, full-scale farming work, pushing a manual lawn mower, shoveling humid and heavy snow, heavy physical labor	None ☐	Once a month or less ☐	2-3 times /month ☐	1-2 times /week ☐	3-4 times /week ☐	5-6 times /week ☐	Every day ☐
		Average duration per day: ( ) minutes					
**Intense exercise**<br>**Examples:** jogging, mountain climbing, playing singles tennis, soccer, skiing, riding a bicycle up a mountain path, intense use of exercise machines	None ☐	Once a month or less ☐	2-3 times /month ☐	1-2 times /week ☐	3-4 times /week ☐	5-6 times /week ☐	Every day ☐
		Average duration per day: ( ) minutes					
Do you currently participate in any regular programs to exercise or improve your physical capacity (exercise club, exercise circle, etc.)?	☐ Yes ☐ No						

Supplementary Figure 2. Back translation of modified Japanese version of cognitively stimulating activity questionnaire

Cognitively stimulating activity

Number：
Date：

Please answer the following questions about your current cognitively stimulating activities.

For each of the cognitively stimulating activities shown below, please indicate the average frequency for the past 12 months using the ☑ mark.							
Cognitively stimulating activities for the past 12 months	None	Once a month or less	2-3 times /month	1-2 times /week	3-4 times /week	5-6 times /week	Every day
**Reading newspapers**<br>**Examples:** Yomiuri, Asahi, Mainichi, Sankei, Chunichi, Hokuriku, Nihon Keizai Shimbun	☐	☐	☐	☐	☐	☐	☐
**Reading magazines**<br>**Examples:** business, sports, art (music, picture, etc.) or hobby (fishing, handicraft, etc.) magazines	☐	☐	☐	☐	☐	☐	☐
**Reading books**<br>**Examples:** novels and nonfiction books	☐	☐	☐	☐	☐	☐	☐
**Playing games**<br>**Examples:** card game, trump, crossword puzzle, sudoku, igo, shogi, Othello and other board games	☐	☐	☐	☐	☐	☐	☐
**Playing music by yourself**<br>**Examples:** playing a musical instrument, singing in a chorus or in a karaoke box, playing music in an orchestra or a band	☐	☐	☐	☐	☐	☐	☐
**Artistic activities**<br>**Examples:** painting with watercolors or oils, carving, calligraphy	☐	☐	☐	☐	☐	☐	☐
**Mental activities**<br>**Examples:** meditation, Zen practice, prayer, sutra chanting	☐	☐	☐	☐	☐	☐	☐
**Handicraft**<br>**Examples:** woodworking, pottery, quilting, sewing	☐	☐	☐	☐	☐	☐	☐
**Group activities**<br>**Examples:** reading session, study meeting, activities in local community	☐	☐	☐	☐	☐	☐	☐
**Social activities**<br>**Examples:** going to a theater, movie, concert, watching a Japanese traditional storytelling including rakugo or bunraku, going out with friends, travelling	☐	☐	☐	☐	☐	☐	☐
**Using a computer (including smartphone)**<br>**Examples:** using the Internet, sending and receiving e-mails, playing computer games, searching on the web, online shopping	☐	☐	☐	☐	☐	☐	☐
	None	Less than one hour	1-2 hours	3-4 hours	5-6 hours	7-8 hours	Over 8 hours
**Television**<br>**How many hours per day do you watch TV?**	☐	☐	☐	☐	☐	☐	☐

Supplementary Figure 3. Distributions of frequencies of physical activity and exercise of forty-eight participants in this study (Examiner A, 1st)

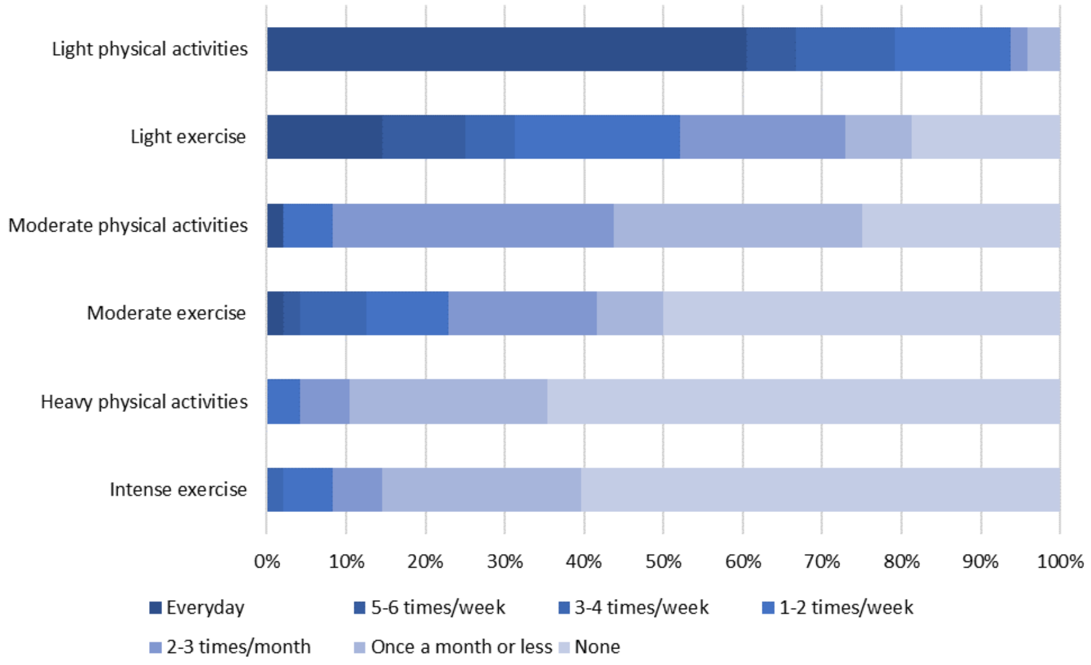

Supplementary Figure 4. Distributions of frequencies of cognitively stimulating activity of forty-eight participants in this study (Examiner A, 1st)

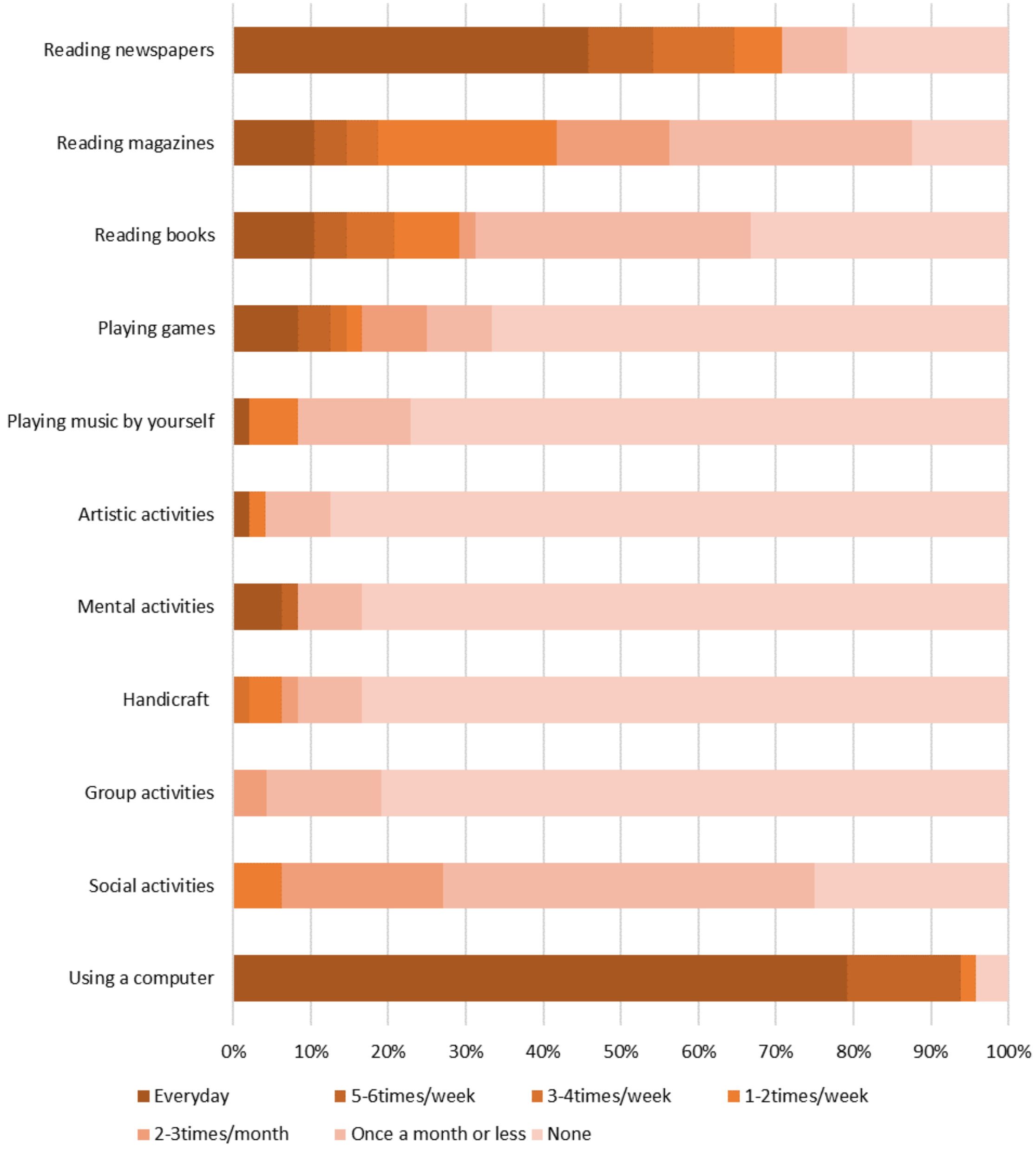

Supplementary Figure 5. Individual Physical Activity Scores of participants

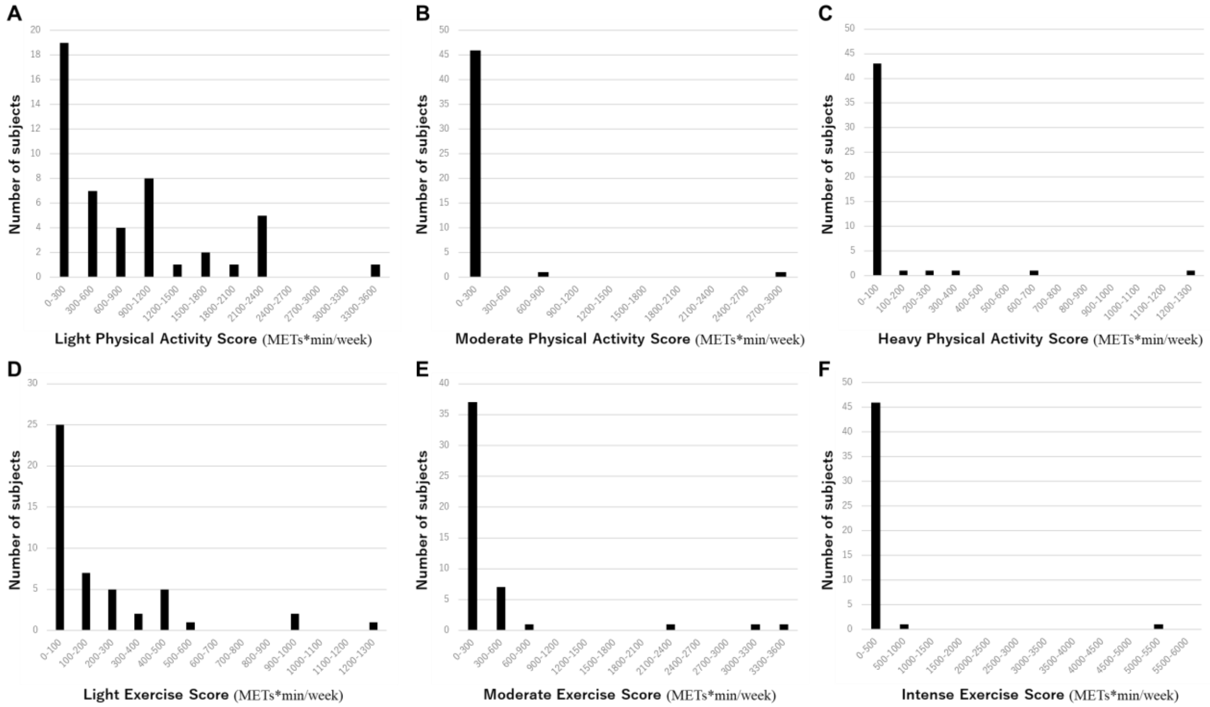

Histograms of Individual Physical Activity Scores (METs*min/week) of forty-eight participants. (A) Light Physical Activity Score, which was calculated as 2.7 (METs) × (the frequency of light physical activities/week) × duration (min). (B) Light Exercise Score, which was calculated as 2.9 (METs) × (the frequency of light exercise/week) × duration (min). (C) Moderate Physical Activity Score, which was calculated as 4.6 (METs) × (the frequency of moderate physical activities/week) × duration (min). (D) Moderate Exercise Score, which was calculated as 5.1 (METs) × (the frequency of moderate exercise/week) × duration (min). (E) Heavy Physical Activity Score, which was calculated as 7.2 (METs) × (the frequency of heavy physical activities/week) × duration (min). (F) Intense Exercise Score, which was calculated as 7.6 (METs) × (the frequency of intense exercise/week) × duration (min).

Supplementary Figure 6. Spearman rank correlation of Inter-rater reliability and test-retest reproducibility of "Total Physical Activity Score" of subjects over 55 years old

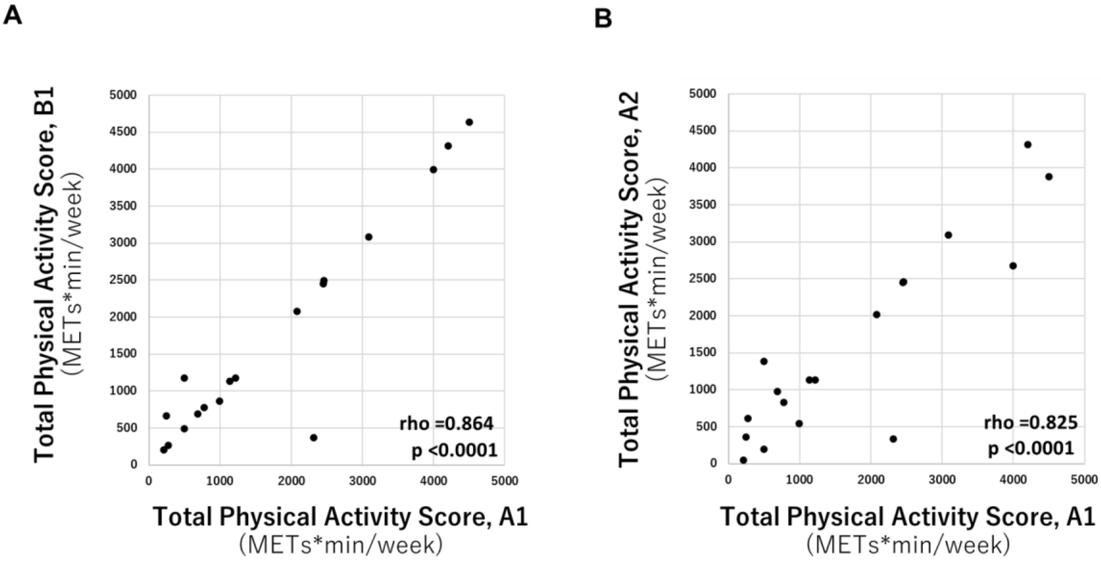

The inter-rater reliability (A) and test-retest reproducibility (B) of "Total Physical Activity Score" of subjects over 55 years old were well retained (rho = 0.864, *p* <0.0001, and rho = 0.825, *p* <0.0001, respectively). A1: examiner A, 1st; B1: examiner B, 1st; A2: examiner A, 2nd.

Supplementary Figure 7. Spearman rank correlation of Inter-rater reliability and test-retest reproducibility of "Total Physical Activity Score" of subjects stratified by female and male

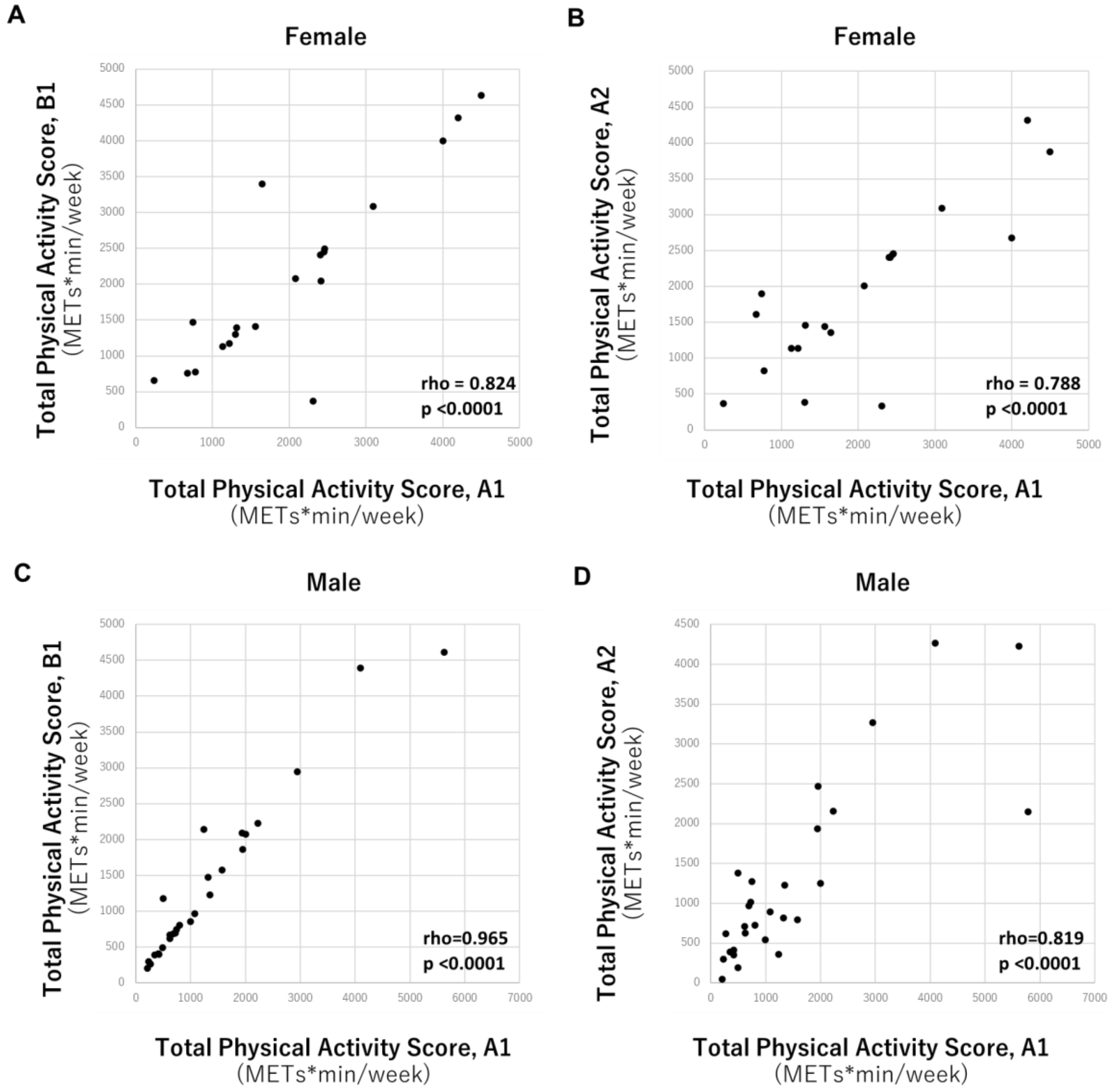

The inter-rater reliability (A) and test-retest reproducibility (B) of "Total Physical Activity Score" of subjects stratified by female and male were well retained. A1: examiner A, 1st; B1: examiner B, 1st; A2: examiner A, 2nd.

Supplementary Table 1. MET values for each activity on the physical activity questionnaire

Light physical activities	Specific Activity#	METs
washing	laundry, fold or hang clothes, put clothes in washer or dryer, packing suitcase, washing clothes by hand, implied standing, light effort	2.0
house cleaning	vacuuming, general, moderate effort	3.3
kitchen work	kitchen activity, general, (e.g., cooking, washing dishes, cleaning up), moderate effort	3.3
bed making	making bed, changing linens	3.3
garbage disposal	dusting or polishing furniture, general	2.5
home maintenance	carpentry, home remodeling tasks, light effort	2.3
simple repair (including car)	home repair, general, light effort	2.5
	Average	2.74

Light exercise	Specific Activity#	METs
slow walking	walking, 2.0 mph, level, slow pace, firm surface	2.8
stretching	stretching, mild	2.3
golfing with a cart	golf, using power cart	3.5
practice on the golf driving range	golf, miniature, driving range	3.0
bowling	bowling	3.0
slow ballroom dancing including waltzing	ballroom, slow (e.g., waltz, foxtrot, slow dancing, samba, tango, 19th century dance, mambo, cha cha)	3.0
	Average	2.93

Moderate physical activities	Specific Activity#	METs
gardening	gardening, general, moderate effort	3.8
DIY	carpentry, general, moderate effort	4.3
window wiping	cleaning, heavy or major (e.g. wash car, wash windows, clean garage), moderate effort	3.5
light snow shoveling	shoveling snow, by hand, moderate effort	5.3
digging a sandbox	digging, spading, filling garden, compositing	5.0
moving furniture	moving furniture, household items, carrying boxes	5.8
wall painting	painting	4.5
	Average	4.60

Moderate exercise	Specific Activity#	METs
fast walking	walking, 3.5 mph, level, brisk, firm surface, walking for exercise	4.3
hiking	hiking or walking at a normal pace through fields and hillsides	5.3
muscle-building	circuit training, moderate effort	4.3
golfing without a cart	golf, walking, pulling clubs	5.3
swimming	swimming laps, freestyle, front crawl, slow, light or moderate effort	5.8
table tennis	table tennis, ping pong	4.0
playing doubles tennis	tennis, doubles	6.0
moderate use of exercise machines	bicycling, stationary, general	7.0
yoga	yoga, Power	4.0
hula dance	ethnic or cultural dancing (e.g., Greek, Middle Eastern, hula, salsa, merengue, bamba y plena, flamenco, belly, and swing)	4.5

	**Average**	**5.05**

**Heavy physical activities**	**Specific Activity[#]**	**METs**
**carrying heavy items**	**carrying heavy loads (e.g., bricks, tools)**	**8.0**
**full-scale farming work**	**farming, vigorous effort (e.g., baling hay, cleaning barn)**	**7.8**
**pushing a manual lawn mower**	**mowing lawn, walk, hand mower**	**6.0**
**shoveling humid and heavy snow**	**snow shoveling, by hand, vigorous effort**	**7.5**
**heavy physical labor**	**manual or unskilled labor, general, vigorous effort**	**6.5**
	**Average**	**7.16**

**Intense exercise**	**Specific Activity[#]**	**METs**
**jogging**	**jogging, general**	**7.0**
**mountain climbing**	**backpacking, hiking or organized walking with a daypack**	**7.8**
**playing singles tennis**	**tennis, singles**	**8.0**
**soccer**	**soccer, casual, general**	**7.0**
**skiing**	**skiing, general**	**7.0**
**riding a bicycle up a mountain path**	**bicycling, mountain, uphill, vigorous**	**8.5**
**intense use of exercise machines**	**circuit training, including kettlebells, some aerobic movement with minimal rest, general, vigorous intensity**	**8.0**
	**Average**	**7.61**

MET values for each activity on the physical activity questionnaire were applied based on a reference.[24]

身体活動

番号：
日付：

現在の身体的活動についての以下の質問に答えてください。

以下に示すそれぞれの身体活動と運動について、最近12か月で、平均してどのくらいの頻度で行ったかを☑で回答してください。またその１日の身体活動時間・運動時間はどのくらいですか。

**軽い身体活動**<br>**例えば：**洗濯、家の掃除、台所仕事、ベッドメイキング、ごみ捨て、家の修繕、（自動車などの）簡単な修理　など	しない ☐	月1回以下 ☐	月に2〜3回 ☐	週に1〜2回 ☐	週に3〜4回 ☐	週に5〜6回 ☐	毎日 ☐
		１日の平均時間　（　　　　）分					
**軽い運動**<br>**例えば：**ゆっくりとした散歩、ストレッチ、カートを用いたゴルフ・打ちっぱなし、ボウリング、ワルツなどのゆっくりとした社交ダンス　など	しない ☐	月1回以下 ☐	月に2〜3回 ☐	週に1〜2回 ☐	週に3〜4回 ☐	週に5〜6回 ☐	毎日 ☐
		１日の平均時間　（　　　　）分					
**中強度の身体活動**<br>**例えば：**庭仕事、日曜大工、窓を拭く、軽い雪かき、砂場を掘る、家具を動かす、壁の塗装　など	しない ☐	月1回以下 ☐	月に2〜3回 ☐	週に1〜2回 ☐	週に3〜4回 ☐	週に5〜6回 ☐	毎日 ☐
		１日の平均時間　（　　　　）分					
**中強度の運動**<br>**例えば：**早歩き、ハイキング、筋力トレーニング、カートを使わないゴルフ、水泳、卓球、テニスのダブルス、エクササイズマシンを中強度使う、ヨガ、フラダンス　など	しない ☐	月1回以下 ☐	月に2〜3回 ☐	週に1〜2回 ☐	週に3〜4回 ☐	週に5〜6回 ☐	毎日 ☐
		１日の平均時間　（　　　　）分					
**強い身体活動**<br>**例えば：**重いものを運ぶ、本格的な農業、手動の芝刈り機を使う、湿って重い雪の除雪、激しい肉体労働　など	しない ☐	月1回以下 ☐	月に2〜3回 ☐	週に1〜2回 ☐	週に3〜4回 ☐	週に5〜6回 ☐	毎日 ☐
		１日の平均時間　（　　　　）分					
**激しい運動**<br>**例えば：**しっかりしたジョギング、山登り、テニスのシングルス、サッカー、スキー、自転車で山道を登る、エクササイズマシンを激しく使う　など	しない ☐	月1回以下 ☐	月に2〜3回 ☐	週に1〜2回 ☐	週に3〜4回 ☐	週に5〜6回 ☐	毎日 ☐
		１日の平均時間　（　　　　）分					
現在、運動するためや体力を向上させるために何らかの定期的なプログラム（運動教室・運動サークルなど）に参加していますか。	☐　はい　　☐　いいえ						

文化活動

番号：
日付：

現在の文化活動についての以下の質問に答えてください。

以下に示す文化活動について、最近12か月で、平均してどのくらいの頻度で行ったかを☑で回答してください。

最近12か月での文化活動	しない	月1回以下	月に2〜3回	週に1〜2回	週に3〜4回	週に5〜6回	毎日
**新聞を読む**<br>**例えば：**読売、朝日、毎日、産経、中日、北陸、日本経済新聞など	☐	☐	☐	☐	☐	☐	☐
**雑誌を読む**<br>**例えば：**ビジネス、スポーツ、芸術(音楽、美術など)、趣味(釣り、手芸など)の雑誌など	☐	☐	☐	☐	☐	☐	☐
**本を読む**<br>**例えば：**小説、ノンフィクションなど	☐	☐	☐	☐	☐	☐	☐
**ゲームをする**<br>**例えば：**カードゲーム、トランプ、クロスワードパズル、数独、囲碁、将棋、オセロ、他ボードゲームなど	☐	☐	☐	☐	☐	☐	☐
**音楽を自分でする**<br>**例えば：**楽器を演奏する、コーラスで歌う、カラオケで歌う、オーケストラやバンドで演奏するなど	☐	☐	☐	☐	☐	☐	☐
**芸術的活動**<br>**例えば：**水彩画を描く、油絵を描く、彫刻をする、習字を書くなど	☐	☐	☐	☐	☐	☐	☐
**精神的活動**<br>**例えば：**瞑想、座禅、お祈り、読経など	☐	☐	☐	☐	☐	☐	☐
**工芸**<br>**例えば：**木工、陶芸、キルティング、裁縫など	☐	☐	☐	☐	☐	☐	☐
**グループでの活動**<br>**例えば：**抄読会、勉強会、地域コミュニティなど	☐	☐	☐	☐	☐	☐	☐
**社会活動**<br>**例えば：**演劇を見に行く、映画に行く、コンサートに行く、落語・文楽などに行く、友人と外出する、旅行など	☐	☐	☐	☐	☐	☐	☐
**コンピュータ（スマホを含む）**<br>**例えば：**インターネットをする、Ｅメールをする、コンピュータゲームをする、ウェブで調べものをする、オンラインで買い物をするなど	☐	☐	☐	☐	☐	☐	☐
	見ない	1時間以下	1〜2時間	3〜4時間	5〜6時間	6〜7時間	8時間以上
テレビ<br>**１日に何時間テレビを見ますか？**	☐	☐	☐	☐	☐	☐	☐

よくある質問(FAQ)とその回答

―身体・文化活動質問票

質問票	質問内容	回答
【身体活動】	「月1回、30分の運動をする」の場合には、時間には「30分」と記入するのか？　それとも、「月1回・30分」を1日の平均にしたら1分になるので、「1分」と記入するのか？	時間には「30分」と記入します。<br>「1 日の平均時間（　）分」のところには、（運動や身体活動を）行った日の時間を書いてください。
【文化活動】	家庭用ゲーム機器とそのソフトウェアによるテレビゲームは「ゲームをする」に、含まれるか？	「ゲームをする」ではなく、「コンピュータ（スマホを含む）」の項目に挙げた例の「コンピュータゲームをする」に含めます。
	スマートフォンの画面上で新聞を読む場合は、「新聞を読む」に該当するのか？	該当します。
	スマートフォンの画面上で「ビジネス雑誌」を読む場合は、「雑誌を読む」に該当するのか？	該当します。
